# Comparable isokinetic quadriceps performance six months after ACL reconstruction with rectus femoris versus hamstring tendon autografts

**DOI:** 10.1002/jeo2.70601

**Published:** 2025-12-17

**Authors:** Márcio Cabral Fagundes Rêgo, Alef Cavalcanti Matias de Barros, Jamilson Simões Brasileiro, Marcelo Cabral Fagundes Rêgo, Camilo Partezani Helito, Carlos Eduardo da Silveira Franciozi, Diego Ariel de Lima

**Affiliations:** ^1^ Hospital Memorial São Francisco Natal Brazil; ^2^ Department of Physiotherapy Federal University of Rio Grande do Norte (UFRN) Natal Brazil; ^3^ Instituto de Ortopedia e Traumatologia, Faculdade de Medicina Universidade de São Paulo (USP) São Paulo São Paulo Brazil; ^4^ Departamento de Ortopedia Universidade Federal de São Paulo (UNIFESP) São Paulo São Paulo Brazil; ^5^ Universidade Federal Rural do Semi‐Árido (UFERSA) Mossoró Brazil

**Keywords:** ACL reconstruction, hamstring autograft, isokinetic dynamometry, quadriceps strength, rectus femoris autograft

## Abstract

**Purpose:**

To compare isokinetic quadriceps performance 6 months after anterior cruciate ligament reconstruction (ACLR) using rectus femoris (RF) versus hamstring tendon (HT) autografts.

**Methods:**

In this cross‐sectional study, 75 male patients who underwent primary ACLR with RF (*n* = 31) or HT (*n* = 44) autografts were evaluated ~6 months postoperatively. Bilateral isokinetic testing at single angular velocity 60°/s (Biodex Multi‐Joint System 4) measured peak torque (PT), PT normalized to body weight (PT/BW), angle at peak torque (aPT), total work (TW), and mean power (PM). Between‐group comparisons used independent t‐tests; paired comparisons were conducted within the RF group.

**Results:**

No between‐group differences were detected for PT, PT/BW, aPT, TW, or PM (*p* > 0.05 for all—primary contrast PT: mean diff. 5.3 Nm; 95% CI –20.4 to 30.9; *p* = 0.689). Within the RF group, the operated limb showed lower PT, PT/BW, TW, and PM than the contralateral limb (all *p* ≤ 0.002), with no difference in aPT.

**Conclusions:**

At 6 months after ACLR, quadriceps performance did not differ between RF and HT autografts. These early data do not address return‐to‐sport timing or graft healing and should not be interpreted as evidence of equivalence (it is not powered for equivalence/non‐inferiority); rather, they support RF as a potential option warranting longer‐term studies incorporating functional tests and patient‐reported outcomes.

**Level of Evidence:**

Level III, cross‐sectional comparative study.

## Graphical abstract

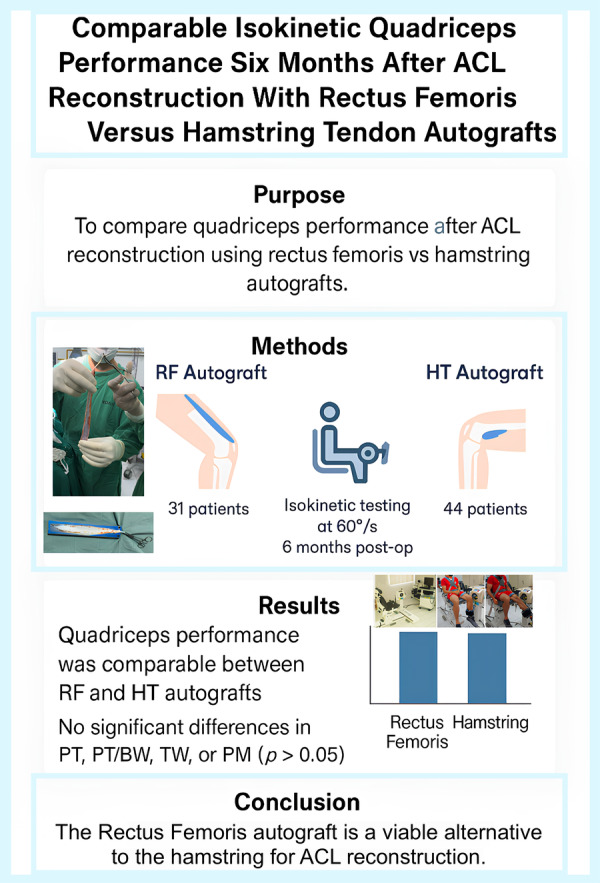

**Abbreviations**
ACL: anterior cruciate ligament
ACLR: anterior cruciate ligament reconstruction
aPT: angle at peak torque
BPTB: bone–patellar tendon–bone
CI: confidence interval
HT: hamstring tendon
IKDC: International Knee Documentation Committee
IRB: institutional review board
KOOS: Knee injury and Osteoarthritis Outcome Score
LSI: limb symmetry index
PM: mean power
PROMs: patient‐reported outcome measures
PT: peak torque
PT/BW: peak torque normalized to body weight
QT: quadriceps tendon
RF: rectus femoris
RTS: return to sport
SD: standard deviation
SPSS: Statistical Package for the Social Sciences
TW: total work

## INTRODUCTION

Anterior cruciate ligament (ACL) reconstruction commonly uses autografts due to their high biocompatibility and superior integration compared to allografts or synthetic grafts [7, 26]. However, donor‐site morbidity remains a concern, especially with grafts harvested from key functional muscles [8, 16].

Among autografts used for ACL reconstruction, hamstring tendons are the most commonly selected worldwide due to their favorable biomechanical properties, ease of harvest, and relatively low donor‐site morbidity. The quadriceps tendon (QT) autograft has recently gained popularity for ACL reconstruction, offering structural strength and versatility in harvesting techniques [3, 13, 30]. A specific technique uses only the superficial layer of the QT—the rectus femoris (RF)—which may reduce morbidity while preserving adequate graft size [5].

Despite its advantages, concerns remain about postoperative quadriceps weakness due to graft harvesting from the extensor mechanism. Importantly, superficial RF harvest is biomechanically distinct from full‐thickness QT harvest, as it preserves deeper fibers of the quadriceps tendon and is therefore expected to result in less pronounced early quadriceps deficits [5]. Several studies have demonstrated that quadriceps tendon autografts—particularly when harvested as full‐thickness grafts—are associated with greater early strength deficits, resulting in more pronounced quadriceps weakness in the early postoperative period compared with hamstring or patellar tendon grafts [4, 17, 18]. Given that quadriceps performance is closely linked to knee function, risk of reinjury, and long‐term osteoarthritis development [24], these deficits raise clinical concerns. Focusing specifically on RF may mitigate such strength loss by preserving deeper fibers and minimizing donor‐site morbidity. This justifies further evaluation of the RF graft, particularly in comparison to the commonly used hamstring tendon [24].

The purpose of this study was to compare isokinetic quadriceps performance between patients undergoing ACL reconstruction with rectus femoris autografts and those with traditional hamstring tendon grafts. It was hypothesized that RF autografts would yield comparable performance.

## MATERIALS AND METHODS

### Study design and ethical approval

This was a cross‐sectional observational study designed to assess quadriceps performance recovery in patients approximately 6 months after undergoing anterior cruciate ligament reconstruction using either an ipsilateral rectus femoris tendon or hamstring tendon autograft.

The study protocol was approved by the institutional review board (IRB protocol no. CAAE: 83875024.9.0000.5294), and all participants provided written informed consent prior to inclusion. All procedures adhered to the ethical standards of the Declaration of Helsinki.

### Patient selection

Patients aged 18 years or older who had undergone primary anterior cruciate ligament reconstruction (ACLR) using either an ipsilateral rectus femoris tendon or a hamstring tendon autograft were eligible for inclusion. All surgeries were performed by the same orthopedic surgical team using standardized techniques. Patients were consecutively recruited between January 2023 and July 2024 during routine postoperative visits. Participants were evaluated at a single time point, approximately 6 months after surgery (between 24 and 28 weeks postoperatively (minimum required follow‐up ≥24 weeks).

Inclusion criteria required patients to have undergone primary ACLR, completed a standardized rehabilitation protocol, and had no prior knee surgery on either side—including contralateral ACLR. Exclusion criteria included multiligamentous injuries, concomitant meniscal or chondral procedures, signs of degenerative joint disease, revision ACLR, or graft harvest from the contralateral limb.

### Graft allocation

Formal randomization was not used. To minimize selection bias, we initially aimed for a consecutive, alternating assignment of graft type (rectus femoris → hamstring → rectus femoris…), as our team is equally experienced with both techniques. This non‐concealed, quasi‐random scheme was subject to change to preserve patient safety and graft adequacy. Participants for the present analysis were recruited consecutively at routine postoperative visits between January 2023 and July 2024, and not all eligible patients consented to participate. These features may introduce allocation and recruitment bias, which we acknowledge in the Limitations.

### Surgical technique

All patients included in the study underwent primary anterior cruciate ligament reconstruction performed by the same orthopedic surgical team, using standardized arthroscopic techniques. No concomitant ligamentous, meniscal, or chondral procedures were performed, as these were part of the exclusion criteria. A pneumatic tourniquet was used on the ipsilateral proximal thigh at approximately 320 mmHg.

#### Graft harvest: Rectus femoris

**Figure 1:**
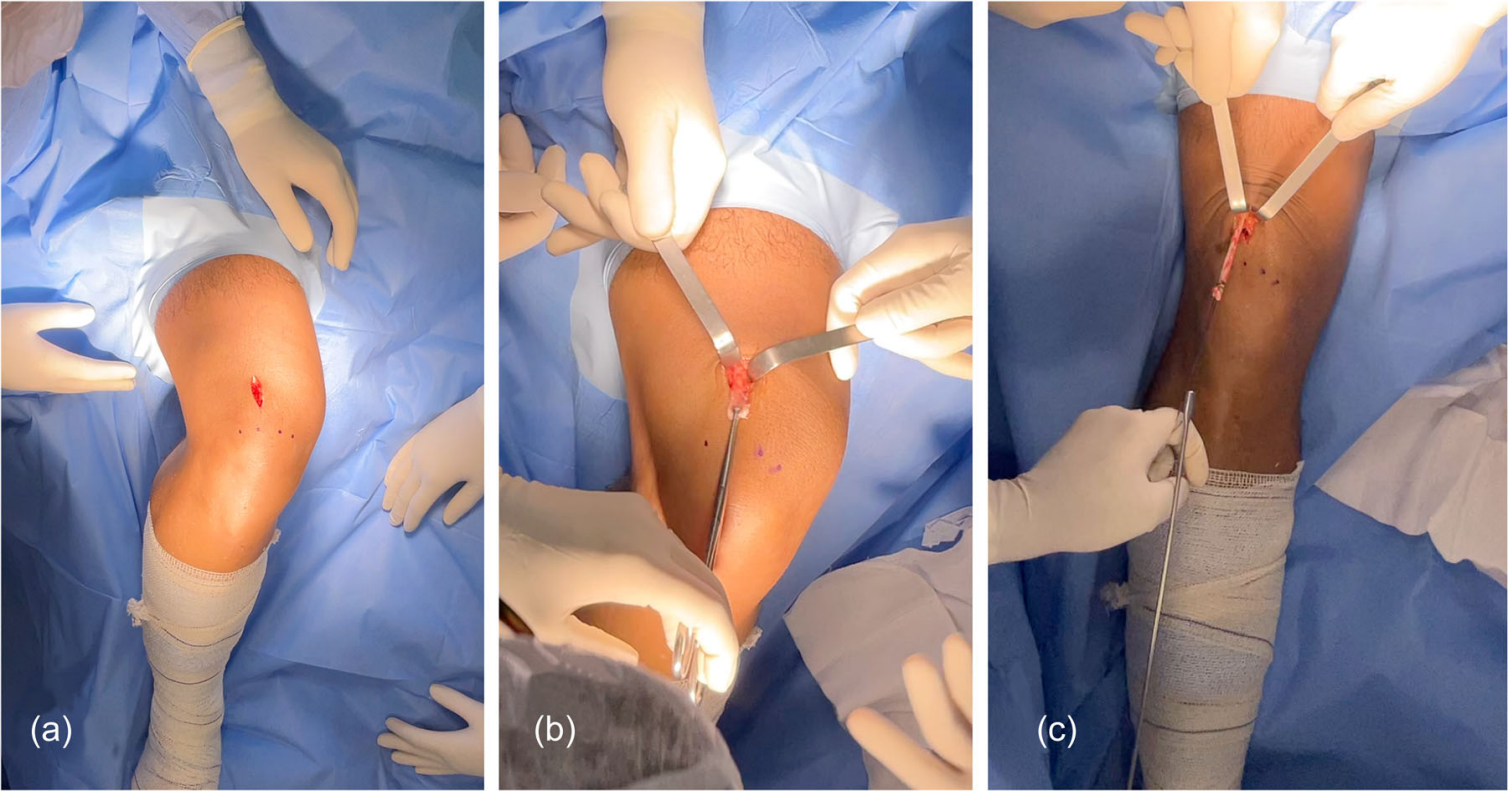
Graft harvest: Rectus femoris. (a) Skin incision over the superior pole of the patella at the junction between the lateral and middle thirds for rectus femoris tendon harvest. (b) A cleavage plane is developed approximately 3 cm proximal to the patella. A 10‐mm‐ wide graft is outlined with two parallel incisions in the superficial layer and detached distally from the patella. The free end is whipstitched with nonabsorbable sutures. (c) The dissection is extended proximally for about 8 cm using scissors, preserving the intermediate and deep layers. With the knee flexed at 20°, the graft is harvested using a closed tendon stripper. Proximally, the rectus femoris lamina broadens; harvest proceeded within this laminar plane using a closed tendon stripper under direct tactile guidance, thereby preserving the intermediate and deep quadriceps tendon layers. No specialized instrumentation was required—once the correct cleavage plane between the rectus femoris and the remaining quadriceps was identified, a standard closed stripper permitted complete proximal release and retrieval of the graft at the desired width.

**Figure 2:**
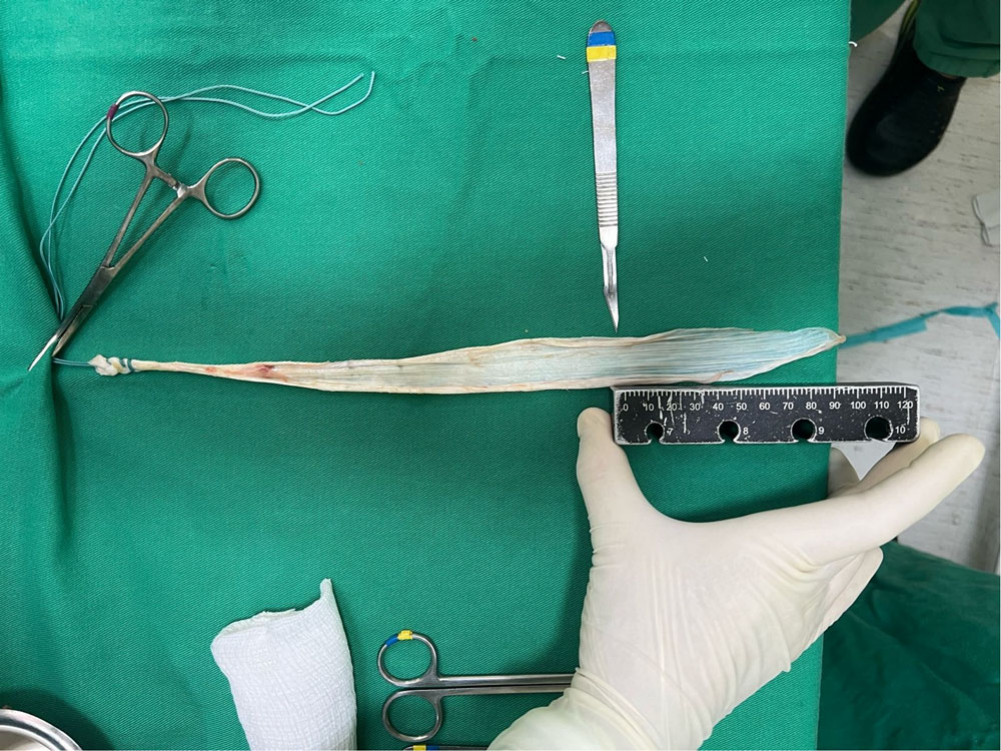
Graft harvest: Rectus femoris. The harvested rectus femoris tendon is placed on the preparation table. Excess muscle fibers and irregularities are removed.

In the RF group, graft harvest followed a previously described technique [5]. A 3‐cm longitudinal incision was made from the superior pole of the patella at the junction of the lateral and middle thirds. After dissection, the quadriceps tendon (QT) was identified, and a 10‐mm‐wide graft was obtained from the rectus femoris (superficial lamina of QT). The dissection extended proximally for approximately 8 cm, preserving the intermediate and deep QT layers. With the knee flexed at 20°, a closed tendon stripper was used to complete the harvest. The distal free end was whipstitched with nonabsorbable sutures. Proximally, the rectus femoris lamina broadens; harvest proceeded within this laminar plane using a closed tendon stripper under direct tactile guidance, thereby preserving the intermediate and deep quadriceps tendon layers. No specialized instrumentation was required—once the correct cleavage plane between the rectus femoris and the remaining quadriceps was identified, a standard closed stripper permitted complete proximal release and retrieval of the graft at the desired width. The graft was folded, with a minimum ACL component length of 9 cm and diameter of 8 mm (mean diameter of 9 mm). The donor site was closed in layers with meticulous hemostasis, and a compressive dressing was applied to minimize the risk of hematoma formation; suction drains were not used (Figures 1 and 2).

#### Graft harvest: The hamstring tendon

In the hamstring tendon (HT) group, grafts were harvested via a 2‐ to 3‐cm incision over the pes anserinus insertion. After opening the sartorius fascia, the semitendinosus and gracilis tendons were identified, dissected, and released distally. A tendon stripper was used to detach the proximal muscular insertions, and the free ends were whipstitched with nonabsorbable sutures. Final graft preparation typically yielded a 4‐strand construct with a mean diameter of 8.0–9.0 mm.

#### ACL reconstruction

ACLR was performed using a standardized arthroscopic technique in both groups. A high anterolateral portal and a low anteromedial portal were created. The femoral tunnel was positioned at the native ACL footprint, located on the lateral femoral condyle posterior to the resident's ridge [22]. A 90° femoral guide was inserted through the anterolateral portal and oriented intra‐articularly at the center of the femoral footprint. Extra‐articularly, the guide was positioned through a 2‐cm longitudinal incision over the lateral femoral epicondyle, with the tunnel directed approximately 4 mm posterior and 8 mm proximal to the epicondyle, corresponding to the anatomic origin of the anterolateral ligament [1, 2]. Importantly, no patients in this study underwent concurrent anterolateral ligament reconstruction or lateral extra‐articular tenodesis; all procedures consisted of isolated ACL reconstructions.

The tibial tunnel was positioned at the native ACL tibial footprint, aligned with the posterior edge of the anterior horn of the lateral meniscus and approximately 15–20 mm anterior to the posterior cruciate ligament [22]. A tibial guide set at 55° was introduced through the anteromedial portal, with its intra‐articular tip centered on the ACL footprint. Extra‐articularly, the guide was positioned 4 cm distal to the tibial joint line, about 1 cm above the pes anserinus insertion and 2 cm medial to the tibial tuberosity. A 2‐mm guide pin was advanced through the guide until visualized intra‐articularly.

Grafts were passed from the tibial to the femoral tunnel using a shuttle suture. Femoral fixation was performed using an interference screw matching the tunnel diameter. Tibial fixation was performed with the knee at 20° of flexion using an interference screw one size larger than the tunnel diameter, ensuring secure tensioning and compression of the graft.

### Postoperative rehabilitation

All patients followed a standardized, time‐based rehabilitation program supervised by the same institutional physical therapy team; the program was identical for both graft groups. Early rehabilitation (postoperative days 0–14) emphasized range‐of‐motion restoration and quadriceps activation, with weightbearing as tolerated and typical discontinuation of crutches by Week 2.

From Weeks 4 to 6, progressive strengthening (closed‐kinetic‐chain) and neuromuscular training were advanced. Between Weeks 6 and 12, lower‐extremity strengthening and proprioceptive training were intensified, with low‐impact cardiovascular conditioning maintained throughout. Straight‐line running was typically introduced between months 4 and 5, followed by light plyometrics and change‐of‐direction drills around months 5–6.

Return to pivoting sports was allowed no earlier than 9 months postoperatively, contingent on completion of sport‐specific functional testing and clinical clearance.

The rehabilitation protocol was identical for both graft groups to minimize variability in recovery outcomes. Neither the physical therapists nor the investigators performing isokinetic testing were blinded to graft type, as the incision site made graft allocation evident.

### Outcome measures and testing protocol

All participants underwent a single‐day isokinetic quadriceps performance assessment conducted in the morning. Bilateral testing was performed in a fixed order: the nonoperative limb was always tested first, followed by the operative limb. Participants were instructed to abstain from alcohol, ergogenic substances, and vigorous physical activity for at least 48 h prior to the evaluation.

**Figure 3:**
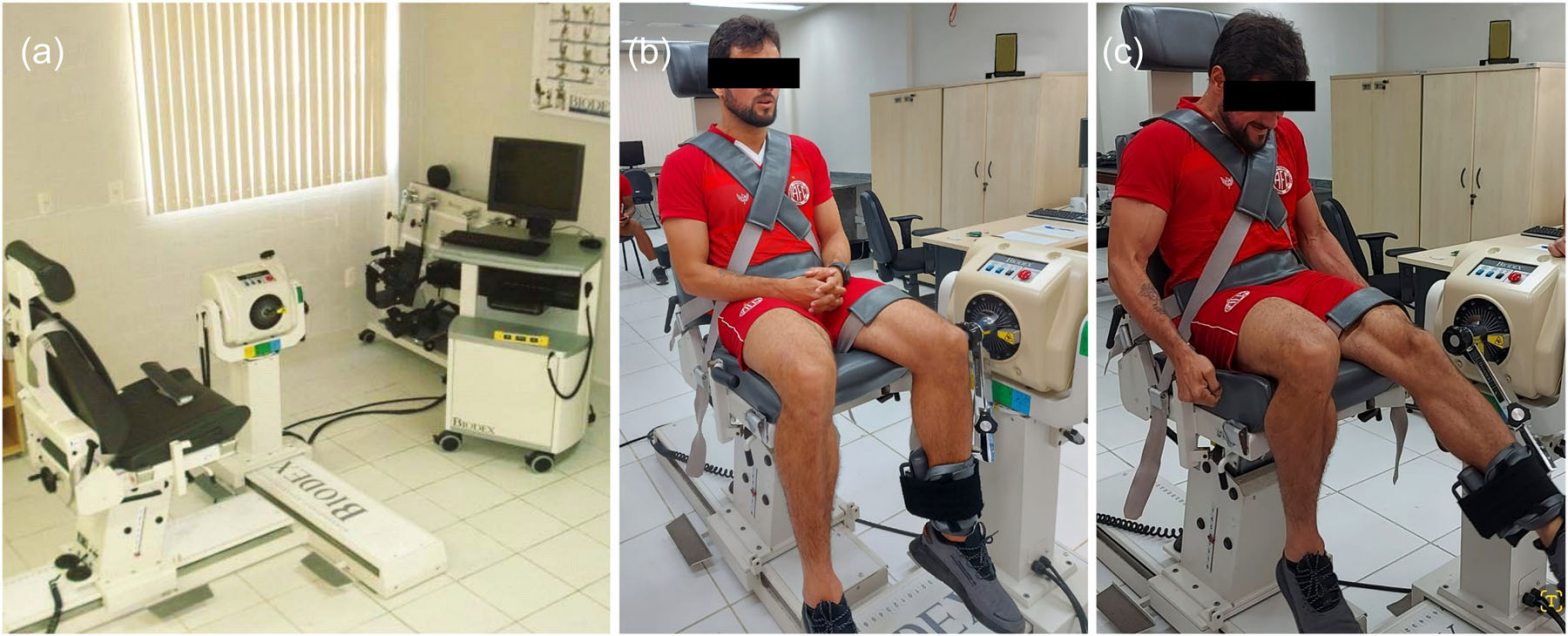
Isokinetic quadriceps performance: (a) Multi‐Joint System 4, Biodex®, USA. (b and c) Participant's isokinetic quadriceps performance during test.

Quadriceps performance was assessed using a computerized isokinetic dynamometer (Multi‐Joint System 4, Biodex®, USA). Measured variables included peak torque (PT), peak torque normalized to body weight (PT/BW), angle of peak torque, total work, and average power [9] (Figure 3).

Before testing, each participant completed a 10‐min self‐paced warm‐up on a stationary bicycle. Subjects were then seated in the dynamometer chair and stabilized with straps across the trunk and pelvis. The knee joint axis was aligned with the dynamometer axis using the lateral femoral condyle as a reference. The resistance arm was positioned and secured 5 cm proximal to the medial malleolus, following the manufacturer's instructions.

For the familiarization phase, participants performed three submaximal concentric contractions of knee flexion and extension at 60°/s, moving from 90° of flexion to full extension. This was followed by five maximal concentric repetitions at the same angular velocity. After a 120‐s rest, the protocol was repeated for the operative limb. Standardized verbal encouragement and real‐time visual feedback were provided throughout the testing session.

### Statistical analysis

Sample size and power: The a priori sample size calculation was based on quadriceps peak torque (PT) as the primary endpoint. An expected standard deviation of 10% was drawn from previous isokinetic performance studies in post‐ACLR athletes [12, 24]. Recruitment was therefore planned to achieve group sizes sufficient to detect clinically meaningful differences within this variability. However, the observed variability in the present study was larger than anticipated, and between‐group effects were small. As a result, the achieved power was insufficient to exclude small effects, and our inference relies primarily on effect sizes and 95% confidence intervals rather than formal equivalence testing.

Analyses were performed in Statistical Package for the Social Sciences (SPSS) version 20.0 (IBM Corp., Armonk, NY, USA). Normality and homoscedasticity were assessed with Shapiro–Wilk and Levene tests, respectively. Between‐group comparisons used independent t‐tests; within‐subject (operated vs. contralateral) comparisons used paired t‐tests. Data are reported as mean ± SD with 95% CIs. We additionally present mean differences and Cohen's *d* to qualify clinical relevance beyond p‐values. Statistical significance was set at *p* ≤ 0.05. Given the small observed between‐group effect (peak torque mean difference 5.3 Nm, 95% CI –20.4 to 30.9; *d* ≈ 0.10) and the larger‐than‐expected SDs, the study is underpowered for small effects; therefore, our inference emphasizes effect sizes and precision rather than equivalence.

## RESULTS

A total of 75 patients participated in the study. Of these, 44 (58.6%) underwent ACL reconstruction using a hamstring tendon (HT) autograft, and 31 (41.4%) received an autograft from the rectus femoris (RF) tendon. All participants were male, with a mean age of 25.6 ± 4.3 years (RF group: 26.1 ± 4.5 years; HT group: 25.3 ± 4.2 years). The majority sustained their injury while practicing pivoting sports (mainly soccer and futsal), and all reported regular preinjury participation in recreational or competitive sports. Although the sample was expanded to increase diversity, no female patients meeting the inclusion criteria presented during the recruitment period, and no sex‐based exclusion criteria were applied.

### Between‐group comparison: Hamstring tendon (HT) versus rectus femoris (RF)

**Figure 4:**
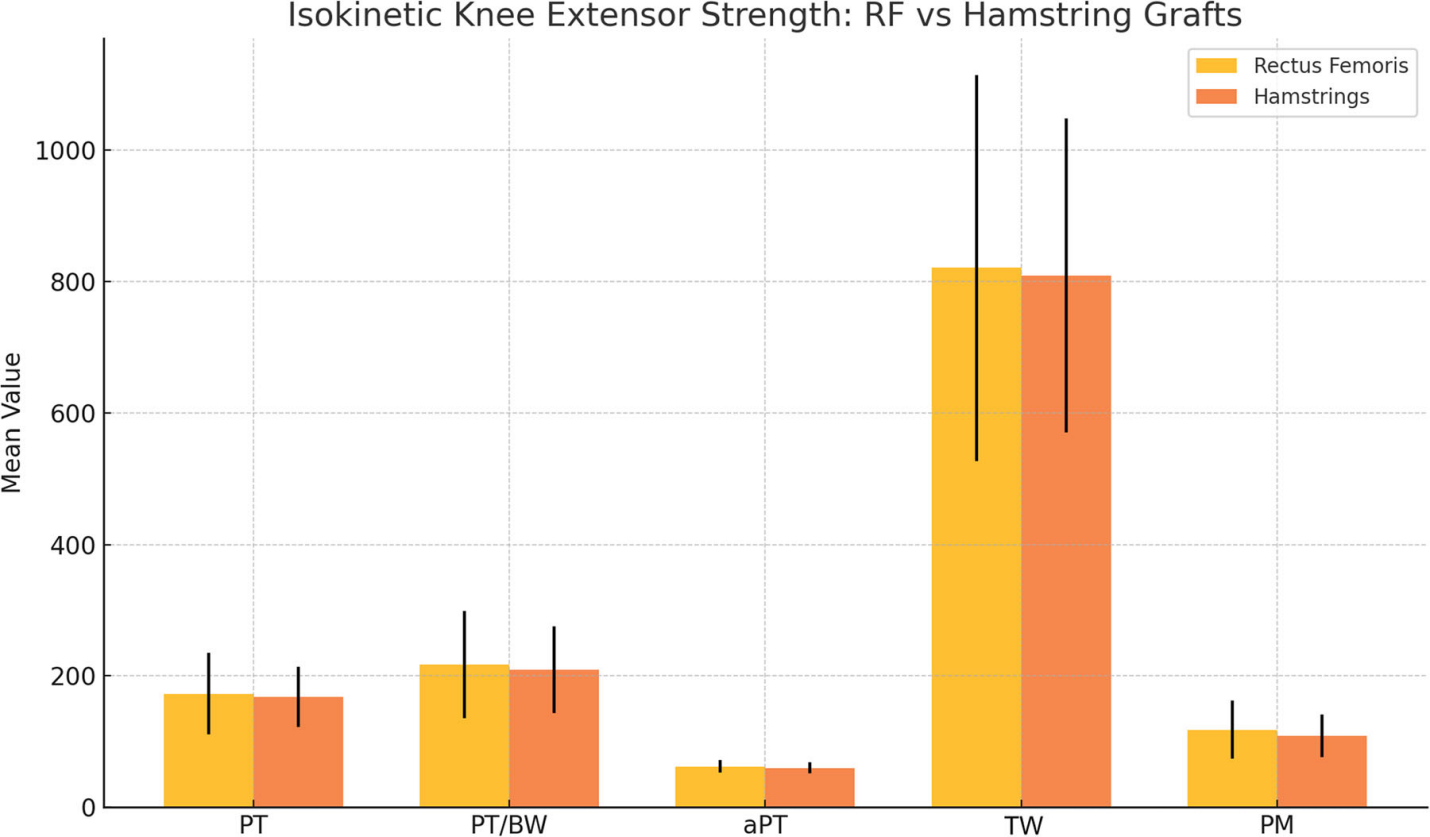
Comparison of isokinetic knee extensor performance between rectus femoris and hamstring tendon graft groups. Bar chart displaying mean values (± standard deviation) for: aPT, angle at peak torque (degrees); PM, mean power (Watts); PT, peak torque (Nm); PT/BW, peak torque normalized to body weight (%); TW, total work (Joules). No statistically significant differences were observed between groups across any variable (*p* > 0.05).

No statistically significant differences were observed between the RF and HT groups in any of the isokinetic knee extensor performance variables. Mean peak torque (PT) was 173.0 ± 62.0 Nm in the RF group and 167.7 ± 45.7 Nm in the HT group (mean difference 5.3 Nm, 95% CI –20.4 to 30.9; *p* = 0.689). Peak torque normalized to body weight (PT/BW) was 217.5 ± 81.4% in the RF group and 209.7 ± 65.8% in the HT group (mean difference 7.8%, 95% CI –26.8 to 42.4; *p* = 0.661). Angle at peak torque (aPT) was 62.2 ± 9.7° in the RF group and 59.9 ± 8.2° in the HT group (mean difference 2.3°, 95% CI –1.9 to 6.5; *p* = 0.280). Total work (TW) was 820.8 ± 294.0 J versus 809.4 ± 239.2 J (mean difference 11.4 J, 95% CI –113.9 to 136.8; *p* = 0.859). Mean power (PM) was 118.1 ± 44.0 W versus 108.6 ± 32.6 W (mean difference 9.5 W, 95% CI –8.8 to 27.7; *p* = 0.314) (Figure 4 and Table 1).

**Table 1:** Comparison of isokinetic knee extensor performance between rectus femoris and hamstring tendon graft groups.

**Variable**	**Rectus femoris (Mean ± SD)**	**Hamstrings (Mean ± SD)**	**95% CI**	***p* value**
Peak torque (Nm)	173.0 ± 62.0	167.7 ± 45.7	–20.4 to 30.9	0.689
Peak torque/body weight (%)	217.5 ± 81.4	209.7 ± 65.8	–26.8 to 42.4	0.661
Angle at peak torque (°)	62.2 ± 9.7	59.9 ± 8.2	–1.9 to 6.5	0.280
Total work (J)	820.8 ± 294.0	809.4 ± 239.2	–113.9 to 136.8	0.859
Mean power (W)	118.1 ± 44.0	108.6 ± 32.6	–8.8 to 27.7	0.314

*Note*: Values are presented as mean ± standard deviation. No statistically significant differences were observed between groups across any variable. Independent samples t‐test was used for all comparisons.

Abbreviations: aPT, angle at peak torque (degrees); CI, confidence interval; PM, mean power (Watts); PT, peak torque (Nm); PT/BW, peak torque normalized to body weight (%); TW, total work (Joules).

### Intragroup comparison (operated vs. non‐operated limb–RF group)

**Figure 5:**
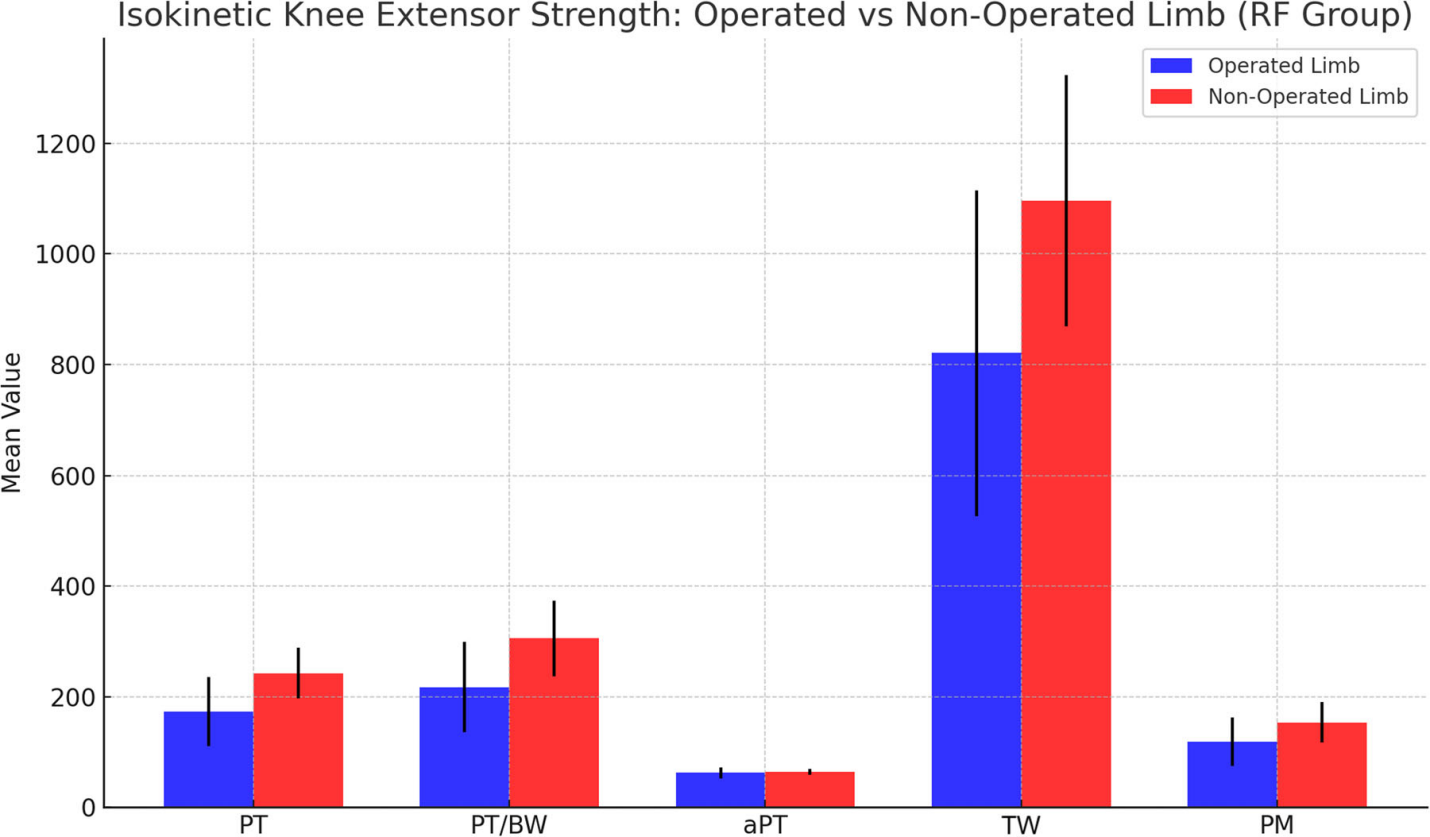
Isokinetic knee extensor performance in operated versus non‐operated limbs in the rectus femoris group. Bar chart displaying mean values (± standard deviation) for: aPT, angle at peak torque (degrees); PM, mean power (Watts); PT, peak torque (Nm); PT/BW, peak torque normalized to body weight (%); TW, total work (Joules). Statistically significant reductions were observed in the operated limb for PT, PT/BW, TW, and PM (*p* < 0.05). Paired t‐test was used for all comparisons.

In the RF group, the operated limb demonstrated significantly reduced extensor performance compared to the contralateral, non‐operated limb. Peak torque was lower in the operated limb (173.0 ± 62.0 Nm) than in the non‐operated limb (242.4 ± 46.1 Nm; mean difference −69.4 Nm, 95% CI −96.6 to −42.2; p < 0.001). PT/BW was also significantly reduced (217.5 ± 81.4% vs. 305.4 ± 68.0%; mean difference −87.9%, 95% CI −125.2 to −50.6; *p* < 0.001). Total work was 820.8 ± 294.0 J in the operated limb versus 1096.8 ± 227.2 J in the non‐operated limb (mean difference −276.0 J, 95% CI −406.8 to −145.2; *p* < 0.001). Mean power was 118.0 ± 44.0 W vs. 153.3 ± 36.7 W, respectively (mean difference −35.2 W, 95% CI −55.4 to −15.1; *p* = 0.002). No significant difference was observed for angle at peak torque (aPT: 62.2 ± 9.7° vs. 63.7 ± 5.5°; mean difference −1.5°, 95% CI −5.4 to 2.4; *p* = 0.457) (Figure 5 and Table 2).

**Table 2:** Isokinetic knee extensor performance in operated versus non‐operated limbs in the rectus femoris group.

Variable	Operated limb (Mean ± SD)	Non‐operated limb (Mean ± SD)	95% CI	*p* value
Peak torque (Nm)	173.0 ± 62.0	242.4 ± 46.1	−96.6 to −42.2	0.001
Peak torque/body weight (%)	217.5 ± 81.4	305.4 ± 68.0	−125.2 to −50.6	0.001
Angle at peak torque (°)	62.2 ± 9.7	63.7 ± 5.5	−5.4 to 2.4	0.457
Total work (J)	820.8 ± 294.0	1096.8 ± 227.2	−406.8 to −145.2	0.001
Mean power (W)	118.1 ± 44.0	153.3 ± 36.7	−55.4 to −15.1	0.002

*Note*: Values are presented as mean ± standard deviation. Statistically significant reductions were observed in the operated limb for PT, PT/BW, TW, and PM (*p* < 0.05). Paired t‐test was used for all comparisons.

Abbreviations: aPT, angle at peak torque (degrees); CI, confidence interval; PM, mean power (Watts); PT, peak torque (Nm); PT/BW, peak torque normalized to body weight (%); TW, total work (Joules).

## DISCUSSION

The main finding of this study is that patients undergoing ACL reconstruction with a rectus femoris tendon autograft demonstrated isokinetic knee extensor performance comparable to those reconstructed with a hamstring tendon autograft at 6 months postoperatively.

This study does not address return‐to‐sport timing or graft maturation at 6 months; it simply shows that, at this early timepoint, rectus femoris and hamstring autografts demonstrated comparable quadriceps performance.

Across all measured variables—peak torque (PT), peak torque normalized to body weight (PT/BW), angle at peak torque (aPT), total work (TW), and mean power (PM)—no statistically significant differences were found between graft types. However, the wide confidence intervals observed indicate limited precision, and small but potentially relevant differences cannot be excluded given the modest sample size. This suggests that the RF graft, despite involving a component of the quadriceps mechanism, does not result in greater extensor strength deficits than the more commonly used hamstring graft [19, 20, 24].

All isokinetic parameters assessed in this study—including peak torque (PT), peak torque normalized to body weight (PT/BW), angle at peak torque (aPT), total work (TW), and mean power (PM)—were statistically similar between the rectus femoris (RF) and hamstring tendon (HT) graft groups. These results reinforce the notion that harvesting the superficial rectus femoris tendon, despite its anatomical contribution to the knee extensor mechanism, does not result in inferior quadriceps performance when compared to the conventional hamstring autograft. This is particularly relevant in clinical scenarios where the use of hamstring tendons is limited, such as in revision surgeries or in patients with previous harvest from the contralateral limb. Our findings align with previous literature suggesting that quadriceps‐based grafts provide comparable functional outcomes to hamstring grafts in terms of strength recovery [11, 24].

Although intergroup comparisons revealed no significant differences, a within‐subject analysis in the RF group demonstrated a substantial quadriceps performance deficit in the operated limb relative to the contralateral, non‐operated side. Significant reductions were observed in peak torque, PT/BW, total work, and mean power, with confidence intervals indicating clinically meaningful differences. These findings suggest that while the RF graft may be comparable to the HT graft at the group level, it is still associated with measurable unilateral extensor weakness. This highlights the importance of interpreting graft‐related performance not only in terms of intergroup comparisons, but also in the context of side‐to‐side functional asymmetry, which has been linked to delayed return to sport and increased risk of reinjury [32]. Moreover, these results provide further evidence supporting the recommendation that return to pivoting or contact sports should ideally occur no earlier than 9 months postoperatively, when strength symmetry is more likely to be achieved [14]. Future studies should therefore incorporate both functional performance tests and PROMs to complement isokinetic assessments and provide a more comprehensive basis for RTS decision‐making.

The unilateral extensor strength deficit observed in our RF group aligns with patterns reported for other graft types. Notably, angle‐specific isokinetic analyses have revealed differences in inter‐limb strength between BPTB and HT graft recipients four to nine months postoperatively, with BPTB patients showing greater extensor torque asymmetry at mid‐range angles compared to HT recipients [15]. Additionally, studies comparing QT, HT, and BPTB grafts have consistently found reduced limb symmetry indices (LSI) in quadriceps performance favoring the contralateral limb at 6–9 months, particularly pronounced in QT and BPTB groups [27]. Thus, our findings of significant quadriceps performance asymmetry in the RF group at 6 months postoperatively are consistent with existing evidence, reinforcing that residual unilateral weakness is common across autograft types, though clinically acceptable symmetry (typically LSI ≥ 90%) often remains elusive within this postoperative timeframe.

Interestingly, no significant differences were observed in the angle at peak torque (aPT), either between graft groups or between operated and non‐operated limbs in the RF group. This finding may indicate that, despite measurable strength deficits, the neuromuscular activation pattern during knee extension remains functionally preserved. The aPT is often interpreted as a surrogate for muscle coordination and timing, and its maintenance suggests that graft harvest from the rectus femoris does not substantially alter the kinematic or neuromotor profile of extensor muscle recruitment. This may help explain the comparable group‐level performance observed between RF and HT grafts, even in the presence of unilateral strength reduction [24, 31]. Because aPT is best interpreted as a surrogate marker of neuromuscular coordination, these findings should be interpreted cautiously. Our results are consistent with prior angle‐specific analyses, suggesting that extensor recruitment patterns may remain functionally preserved despite strength deficits.

Despite theoretical concerns regarding quadriceps dysfunction following harvest of the RF tendon, our findings indicate that its impact on knee extensor performance is not inferior to that of hamstring tendon grafts at 6 months postoperatively. This supports the growing body of evidence that positions the quadriceps tendon (QT)—including its superficial RF component—as a viable graft source for ACL reconstruction. Biomechanical studies have shown that QT autografts have favorable structural properties, including a greater cross‐sectional area compared to both hamstring and bone–patellar tendon–bone (BPTB) grafts [6, 24, 25]. Clinical outcomes have also been reported to be comparable in terms of knee stability, functional recovery, and graft failure rates when QT is compared to HT and BPTB grafts [4].

A key advantage of the QT graft lies in its versatility. It can be harvested in partial‐ or full‐thickness configurations, with or without a bone block. Even with anatomical variability, the superficial layer of the rectus femoris can be harvested reliably before merging with deeper layers [29]. Moreover, soft tissue‐only QT autografts appear to result in fewer donor‐site complications [10]. Concerns regarding donor‐site hematoma and transient extensor strength deficits do exist, but reports suggest that quadriceps recovery following QT harvest is similar to that of BPTB [18].

The clinical interest in using the rectus femoris tendon has grown in recent years. Authors such as Raman et al. [21], Thamrongskulsiri et al. [28], Fink et al. [13], Rego et al. [23] and Barroso et al. [5] have described its successful application, particularly in complex or multiligament reconstructions, due to its length and adaptability. Given these advantages, the RF graft appears to be a viable and increasingly recognized option. Future studies should explore long‐term functional outcomes, sport‐specific recovery, and inclusion of female athletes to better define the role of RF grafts in contemporary ACL surgery.

### Limitations

This study has several limitations. (1) single timepoint (6 months), prone to arthrogenic inhibition and high variability; (2) non‐randomized graft allocation with consecutive recruitment at routine visits, introducing potential selection bias; (3) all‐male cohort, limiting generalizability; (4) single angular velocity (60°/s), not capturing endurance/high‐speed function; (5) absence of PROMs/functional tests; and (6) underpowered between‐group contrasts given the observed variability (e.g., PT difference 5.3 Nm; 95% CI −20.4 to 30.9; very small effect size, low achieved power). Additionally, only isokinetic knee quadriceps performance was evaluated, without complementary assessments of neuromuscular control or return‐to‐sport criteria. Finally, the lack of preoperative baseline data and standardized symmetry indices (e.g., limb symmetry index) limits comparisons with other studies. Further limitations include the absence of blinding of therapists and assessors and lack of preoperative baseline strength values.

## CONCLUSION

At 6 months after ACLR, quadriceps performance did not differ between RF and HT autografts. These early data do not address return‐to‐sport timing or graft healing and should not be interpreted as evidence of equivalence (it is not powered for equivalence/non‐inferiority); rather, they support RF as a potential option warranting longer‐term studies incorporating functional tests and patient‐reported outcomes.

## AUTHOR CONTRIBUTIONS

All authors made substantial contributions to the conception and design of the study, data acquisition and interpretation, drafting or critically revising the manuscript, and approved the final version for publication. Specifically: Diego Ariel de Lima: Study conception, data analysis, manuscript drafting, and supervision. Márcio Cabral Fagundes Rêgo and Marcelo Cabral Fagundes Rêgo: Surgical procedures, data collection, and clinical follow‐up. Alef Cavalcanti Matias de Barros and Jamilson Simões Brasileiro: Isokinetic testing design and data analysis. Camilo Partezani Helito and Carlos Eduardo da Silveira Franciozi: Methodological review and manuscript revision. All authors read and approved the final manuscript.

## CONFLICT OF INTEREST STATEMENT

The authors declare no conflicts of interest.

## ETHICS STATEMENT

This study was approved by the Institutional Review Board of Universidade Federal Rural do Semi‐Árido (UFERSA) (CAAE: 83875024.9.0000.5294) and conducted in accordance with the Declaration of Helsinki. Written informed consent was obtained from all participants prior to inclusion in the study.

## Data Availability

The data sets generated and analyzed during the current study are available from the corresponding author on reasonable request.
